# Bibliography

**Published:** 1891-05

**Authors:** 


### BIBLIOGRAPHY.

HEREDITY. HEALTH AND PERSONAL BEAUTY. By JOHN V. SHOEMAKER, A.M., M.D. F. A. Davis, Publisher. Philadelphia, 1890.

It is somewhat singular that there is a growing tendency on the part of professional men to step outside the legitimate pathway to which they have been trained to walk and seek recreation, it may be in by-ways, not wholly their own, and oftentimes entirely foreign to what might be supposed their ordinary thoughts.

This work by Dr. Shoemaker, while in many directions in harmony with medical studies, in others it is quite evident that the author has trespassed on unfamiliar ground, yet notwithstanding this, there is throughout manifested a desire to rouse the non-scientific to higher conceptions of duty.

He deals with many subjects in the four hundred and twenty-two pages of this unique book, too many to even quote the titles.

We are tempted to transfer many passages, but space will not permit, nor would an extended notice be in accordance with the objects of this journal, but room must be found for this exquisite description of a farm-house recognizable by every one: "Who does not know the stuffy, darkened rooms of the ordinary farm-house, the subtle smell in the chamber of the painted window-shades, and of the long-plucked feathers in the pudding bed; the sitting-room with its single ray of light, sparing the colors of the carpet, by which one navigates towards a book; and the one room devoted to refreshments, where alone are light and air, and flies hold high revelry?"

The chapters on "The Regulative Law of Life and Growth" and "The Bath as Promotive of Health and Beauty" claim the special attention of the reader. The author is something of a traveller, and views the world with cosmopolitan eyes, enlivening his pages by scraps of information gathered here and there, adding materially to the freshness and interest of the work.

Probably the best that can be said of this book is that the writer began its examination by a general review, but found it so full of excellent thoughts that the interest was held in reading chapter by chapter to the end.

